# Neonatal Mucosal Immune Stimulation by Microbial Superantigen Improves the Tolerogenic Capacity of CD103^+^ Dendritic Cells

**DOI:** 10.1371/journal.pone.0075594

**Published:** 2013-09-27

**Authors:** Anna Stern, Agnes E. Wold, Sofia Östman

**Affiliations:** Department of Infectious Medicine, Institute of Biomedicine, Sahlgrenska Academy at the University of Gothenburg, Gothenburg, Sweden; MRC National Institute for Medical Research, United Kingdom

## Abstract

Food allergy represents failure to develop tolerance to dietary proteins. Food allergy has increased in prevalence in parallel with decreased exposure to microbes during infancy. In mice, neonatal peroral exposure to the strongly T cell stimulating superantigen staphylococcal enterotoxin A (SEA), enhances the capacity to develop oral tolerance to a novel antigen encountered in adult life. A population of antigen-presenting cells in the gut, the CD103^+^ dendritic cells (DCs), is thought to be involved in oral tolerance development, as they convert naïve T cells into FoxP3^+^ regulatory T cells (Treg). This function depends on their capacity to convert vitamin A to retinoic acid, carried out by the retinal aldehyde dehydrogenase (RALDH) enzyme. Here, newborn mice were treated with superantigen and DC function and tolerogenic capacity was examined at six weeks of age. We observed that, in mice fed superantigen neonatally, the CD11c^+^ DCs had increased expression of RALDH and *in vitro* more efficiently induced expression Foxp3 expression to stimulated T cells. Further, these mice showed an accumulation of FoxP3^+^ T cells in the small intestinal lamina propria and had a more Ag-specific FoxP3^+^ T cells after oral tolerance induction *in vivo*. Moreover, the improved oral tolerance, as shown by increased protection from food allergy, was eradicated if the Vitamin A metabolism was inhibited. These observations contribute to the understanding of how a strong immune stimulation during the neonatal period influences the maturation of the immune system and suggests that such stimulation may reduce the risk of later allergy development.

## Introduction

Bacterial colonization of the intestine starts after birth and leads to activation of the immune system. The hygiene hypothesis states that decreased exposure of microbes during infancy leads to defective maturation of the immune system and results in increased risk of developing allergies [1], [2]. This is supported by experimental data, in germ free mice, showing that the absence of microbes results in an immature immune system and defective capacity to develop oral tolerance [3]–[5].

Oral (or mucosal) tolerance is the normal response of the gut immune system to novel harmless protein antigens [6]. The mechanism of oral tolerance is not completely understood, but it is suggested that dietary antigens are carried by DCs from the gut mucosa to the draining mesenteric lymph nodes (MLN) where they are presented to naïve T cells in a manner that leads to their conversion into induced Tregs [7]. Tregs are CD4^+^ T cells that express the transcription factor FoxP3 and suppress immune responses both by controlling the activation of CD4^+^ T helper cells and by de-activating APCs. Increased level of FoxP3^+^ Tregs in the gut mucosa accompanies the induction of oral tolerance [7]. Furthermore, APCs in the GALT are known to be more prone to present an antigen in a tolerogenic, rather than immunogenic fashion and oral tolerance has been linked to presentation of dietary antigens by such “tolerogenic” DCs [8], [9]. A population of DCs expressing CD103 is found in the GALT of both mice and humans [10], [11]. They convert naïve T cells, to which they present their antigens, into FoxP3^+^ Tregs. This action depends on the capacity of CD103^+^ DCs to convert Vitamin A (retinol) to retinoic acid, a reaction catalyzed by the enzyme RALDH, also called ALDH1A. Furthermore, CD103^+^ DCs imprint gut homing properties to T cells, also depending on retinoic acid production, i.e. a functional RALDH [11]–[13].

How microbes regulate tolerance to innocuous antigens, and whether certain microbes are better than others in this respect, have not been determined. We have previously reported, in Swedish children, that neonatal colonization by *Staphylococcus aureus* (*S. aureus*) is inversely related to the risk of developing food allergies [14]. *S. aureus* strains can produce one or several toxins with superantigenic function, including staphylococcal enterotoxins (SE) A, B, C, D and E, as well as toxic shock syndrome toxin-1 (TSST-1). Superantigens are the strongest known T cell stimulants. They bind to MHC class II molecules on APCs, linking them to T cells of one or a few Vβ TCR subsets. Infants spontaneously colonized with superantigen-producing *S. aureus* strains in the gut had higher serum levels of IgA, than infants colonized by non-superantigen producing *S. aureus* strains or not colonized by *S. aureus*, demonstrating the strong immune activation properties of superantigens. Furthermore, high serum IgA levels were connected to lower risk of eczema in the examined cohort [15].

In order to investigate if exposure to superantigen in the neonatal period could modulate the maturation of the immune system, we set up a model where we gave newborn mice peroral doses of SEA during the first two weeks of life. We found that mice that were exposure to SEA as neonates had an improved ability to become orally tolerized as adults, which was demonstrated as enhanced protection in an airway allergy model [16]. Neonatally SEA exposed mice also displayed an increased expression of gut homing markers on Tregs [16]. Since this homing phenotype is imprinted on naïve T cells by CD103^+^ DCs, and because these cells also mediate Treg conversion, we here wanted to investigate the function of the CD103^+^DC subset in adult mice that had been treated with superantigen as neonates. As infants spontaneously colonized with *S. aureus* were protected from food allergy, we also wanted to examine whether improved oral tolerance due to experimental superantigen exposure would confer improved protection in a food allergy model.

## Methods

### Animals

Male and female BALB/c mice (6-week-old) and male Sprague-Dawley rats (12-week-old) were purchased from Charles River (Sulzfeld, Germany). BALB/c and DO11.10 OVA TCR transgenic mice , whose CD4^+^ T cell receptor recognizes the immunodominant epitope OVA_323–339_ [17], were bred and housed at the animal facility of the University of Gothenburg. Animal care was in accordance with institutional guidelines.

This study was carried out in strict accordance with recommendations from the Swedish board of agriculture and approved by the regional Ethics committee (Gothenburg Counties of Västra Götaland and Värmland, Kammarrätten i Göteborg, Box 1531, 401 50 GÖTEBORG, Permit No: 229-2010).

### Experimental design

**Figure 1:**
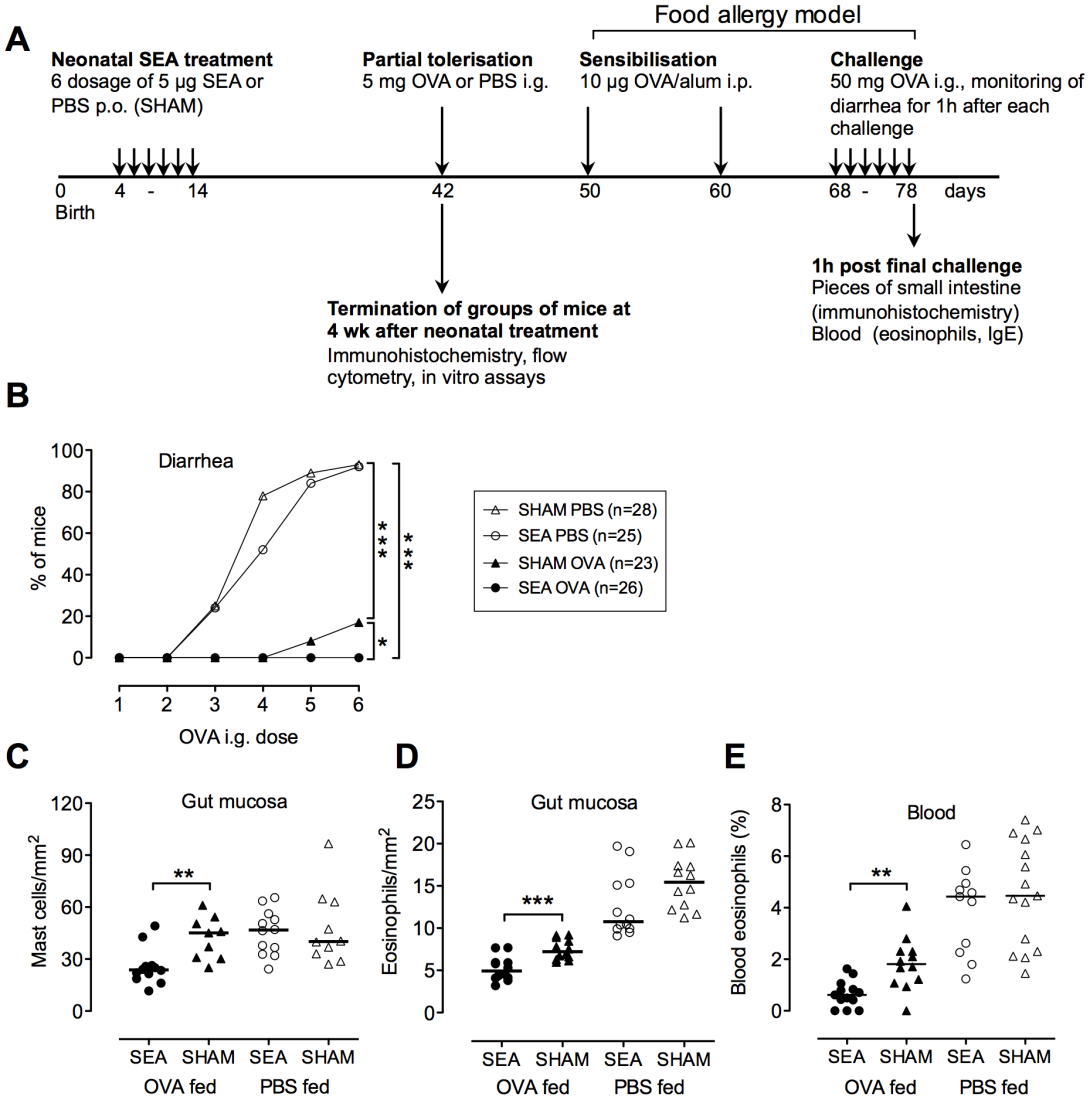
Neonatal treatment with SEA enhances the capacity to develop oral tolerance, manifested as increased protection in a food allergy model. (A) Experimental design; Newborn BALB/c pups were perorally exposed to staphylococcal enterotoxin A (SEA) every other day during two weeks. SHAM-treated mice received PBS. Four weeks later, the mice were sacrificed and mesenteric lymph nodes (MLN) and gut sections were examined. Other mice received 5 mg OVA to induce oral tolerance, or PBS as control. Food allergy was induced by sensitization through two i.p. injections of alum-adsorbed OVA followed by i.g. challenge with OVA. Hypersensitivity to OVA was measured as (B) onset of diarrhea, (C) infiltration of mast cells and (D) eosinophils into the gut mucosa and (E) increased levels of eosinophils in the blood. In B, each symbol represents percentage of diarrhea positive mice in the group (n = 23–28) after each OVA challenge. In C–D each symbol represents one animal (n = 8–15) and horizontal line shows the median value for the group. Data shown are pooled from three experiments * P<0.05, ** P<0.01 and *** P<0.001, analyzed with Fisher's exact test (onset of diarrhea) or Mann-Whitney U-test.

The experimental design is shown in Figure 1. Pups were given 5 µg of SEA perorally (Sigma-Aldrich, St. Louis, MO), in 5 µL PBS, or PBS alone (SHAM), every other day starting when the pups were 4–5 days old (totally 6 times during the first 2 weeks of life). The mice were rested until 6 weeks of age, when they were either sacrificed, for examination of the small intestinal mucosa (immunohistochemistry) and MLN (flow cytometry), or orally tolerized to OVA and further tested for tolerance development in the food allergy model.

### Oral tolerance protocol

At 6 weeks of age, mice were fed OVA. The dose was selected to achieve partial tolerance; a single dose of OVA (5 mg grade V, Sigma-Aldrich) in 300 µL PBS was given by gavage; controls received 300 µL PBS. The degree of tolerance was tested in an OVA food allergy model (see below). To reduce Vitamin A metabolism, groups of mice were given the RALDH inhibitor Citral (Sigma-Aldrich) in the drinking water (2 mg/mL, approximately 8 mg/mouse per day) at the time for tolerization; Citral treatment began 10 d prior to the OVA feed and continued for another 4 days. Controls received normal drinking water. After Citral was removed, mice were either sacrificed for flow cytometric analysis of MLN cells or subjected to the food allergy protocol, as described below.

### OVA-induced food allergy model

The degree of tolerance to OVA was tested in the OVA-induced food allergy model (Fig. 1) [18]. Starting 8 days after partial oral tolerization, mice were sensitized twice with 10 µg OVA adsorbed onto 2 mg of Al(OH)_3_ gel (Alum; Sigma-Aldrich) by i.p. injections. Hypersensitivity was evoked by challenge, i.e. repeated i.g. deposition of 50 mg OVA in 300 µL PBS (every other day, totally 6 times) (Fig. 1). Mice were deprived of food for 2 h prior to each challenge dose. Diarrhea was assessed by visually monitoring mice for up to 1 h after challenge. Mice demonstrating profuse liquid stools were recorded as positive. One hour after the last OVA challenge, the mice were sacrificed.

Blood was collected for analysis of total and OVA-specific IgE in serum. Pieces of the small intestine were excised, fixed in 4% formaldehyde for 24 h, washed in PBS overnight and stored in 70% ethanol until being embedded in paraffin, sectioned (4 µm) and stained with toluidine blue for visualization of total cell infiltration or with May Grünwald/Giemsa for enumeration of mast cells and eosinophils (Histocenter, Gothenburg, Sweden). The assessment of stained cells was carried out under a Leica Q500MC microscope (Leica, Cambridge, UK). The numbers of mast cells were counted in 3 cross-sections of the small intestine (40× lens) and the area of each section was estimated using BioPix IQ 2.0 software (Biopix AB, Gothenburg, Sweden). Total cell infiltration and eosinophils were counted in 9 randomly selected villi from 3 separate cross-sections of the small intestine (20× lens). Number of positively stained cells and villus area were estimated using BioPix IQ 2.0 software. For enumeration of blood eosinophils, blood was drawn into EDTA tubes (Sarstedt), cells were pelleted by centrifugation and red blood cells were lysed with NH_4_Cl buffer. Remaining cells were centrifuged onto glass slides (Cytospin) and stained with May Grünwald/Giemsa. The proportion of eosinophils among at least 100 cells was calculated.

### Determination of OVA specific and total IgE

OVA-specific IgE was assayed by passive cutaneous anaphylaxis. Sprague-Dawley rats were anaesthetized by i.p. injection of xylazine (8 mg/kg) and ketamine (40 mg/kg). Test mouse sera were serially diluted in twofold steps with PBS and 50 µL of each dilution were injected intradermally into the shaved dorsal skin of the rats. After 72 h, the rats were challenged i.v. with 5 mg OVA in 1 mL PBS with 1% Evans blue (Sigma-Aldrich). Half an hour later, the rats were sacrificed and the skin was examined. The titer was defined as 1/the highest dilution giving a blue spot of ≥5 mm in diameter.

Serum IgE concentration was determined by sandwich ELISA. Costar-plates were coated with capture antibody (mouse anti-IgE antibody, 1 µg/mL; BD Biosciences, San Jose, CA). Biotinylated anti-mouse IgE (2 µg/mL; BD Biosciences) was used as the detection antibody. Serum was diluted 3-fold in 4 steps and standard was diluted twofold in 7 steps, starting with 100 ng/mL. The limit of detection was 6 ng/mL.

### Antigen-specific response after oral tolerance induction

At 6 weeks of age, neonatally SEA- or SHAM-treated mice were injected i.v. with 1×10^6^ OVA-specific CD4^+^ T cells, isolated by magnetic bead sorting (CD4^+^ T cell selection kit II, Miltenyi, Bergisch Gladbach, Germany), from the spleens of DO11.10 mice. 24 h after injection of OVA-specific T cells, mice were fed 5 mg OVA or PBS and at 10 h later mice were sacrificed and the MLNs collected. MLN cell suspensions were prepared and analyzed by flow cytometry for the presence of OVA-specific KJ1.26^+^ CD4^+^ T cells. The expression of FoxP3 was determined among the KJ1.26^+^ CD4^+^ T cells. Cells were acquired on a FACSCantoII (BD Biosciences) and analyzed with FlowJo software (Treestarinc., Ashland, OR).

### Immunohistochemistry of gut mucosa

The proximal small intestine (approx. 7 cm) was excised, fixed in 4% formaldehyde for 24 h, washed in PBS overnight and stored in 70% ethanol until being embedded in paraffin. Tissue sections (4 µm) were prepared (Histocenter, Gothenburg, Sweden) and stained with antibodies against FoxP3 (clone: FJK-16s; ebioscience, San Diego, CA), TGF-β (ab66043; Abcam, Cambridge, UK) or CD3 (Clone: 145-2C11; BD bioscience), with toluidine blue for visualization of total cell infiltration, or with May Grünwald/Giemsa for enumeration of mast cells and eosinophils (Histocenter, Gothenburg, Sweden). The assessment of stained cells was carried out under a Leica Q500MC microscope (Leica, Cambridge, UK). Positively stained cells were counted in 9 randomly selected villi from 3 separate cross-sections of the small intestine (20× lens) and numbers of cells and villus area were estimated using BioPix IQ 2.0 software.

### Antibodies used for flow cytometry

Cells from MLN or *in vitro* cultures were stained for surface and intracellular expression according to standard procedures. The following antibodies were used; DO11.10 TCR (clone: KJ1.26), CD4 (clone: RM4-5), FoxP3 (clone: FJK-16s), CD19 (clone: 1D3), α4β7 (clone: DATK32), CD11c (clone: HL3), CD103 (clone: M290), all purchased from BD Bioscience. To measure the expression of ALDH, the ALDEFLUOR® fluorescent reagent system was used, followed by surface staining of CD11c-APC and CD103-PE, according to the manufacturer's instructions (Stemcell technologies, Grendoble, France). All cells were acquired using FACSCantoII (BD Biosciences) and analyzed with FlowJo software (Treestarinc., Ashland, OR).

### CDllc^+^ DC- T cell co-cultures

CD11c^+^ cells were enriched from MLN or spleen cell suspensions, from neonatally SEA or SHAM treated mice, by CD11c^+^ magnetic bead sorting, according the manufacturer's instructions (Miltenyi, BergischGladbach, Germany). CD11c^+^DCs (5×10^4^/well) were pulsed with OVA (250 µg/mL) for 2 h at 37°C and co-cultured with OVA-specific T cells (5×10^4^), purified from the spleen of DO11.10 mice by negative selection using magnetic beads according to the manufacturer's instructions (Miltenyi) and stained with Carboxyfluorescein succinimidyl ester (CFSE) according the manufacturer's instructions (Invitrogen, Oregon, USA). After 6 d of co-culture in Iscove's medium supplemented with 2 mMl-glutamine, 50 µM mercaptoethanol, 50 µg/ml gentamycin, 10% fetal calf serum, DO11.10 T cells were analyzed by flow cytometry for expression of surface CD4 and α4β7, according to standard protocols. Proliferation was estimated as the proportion of cells with reduced staining intensity of CFSE.

CD11c enriched DCs, see above, from MLN of SEA and SHAM treated mice, were stained with CD103-PE antibody and FACS sorted into CD11c^+^CD103^+^ and CD11c^+^CD103^neg^ fractions. The sorted CD103^+^and CD103^neg^ cells (5×10^4^/well) were pulsed and cultivated with OVA-specific T cells, as described above. For culture, Iscove's medium supplemented with 2 mM-glutamine, 50 µM mercaptoethanol, 50 µg/ml gentamycin, 10% fetal calf serum and All trans Retinol 100 nM (Sigma, Steinheim, Germany) was used. After 6 d of co-culture, DO11.10 T cells were analyzed by flow cytometry for intracellular expression of FoxP3 and surface CD4 and α4β7, according to standard protocols.

### Statistical analysis

Statistical analysis was conducted using the nonparametric Mann-Whitney U-test or Fisher's exact test (diarrhea onset) or two-way ANOVA followed by Bonferroni post test. *P* values<0.05 were considered statistically significant.

## Results

### Neonatal SEA treatment improves oral tolerance, as demonstrated by protection in a food allergy model

We have previously reported that feeding the superantigen SEA during the first two weeks of life enhances the capacity to develop tolerance to OVA fed at six weeks of age. Tolerance was measured as enhanced protection in the OVA-airway allergy model [16]. We here wanted to investigate whether such improvement in oral tolerance would also confer protection against OVA-mediated food allergy. The experimental design is shown in figure 1A. Newborn pups were perorally exposed to the *S. aureus* superantigen SEA every second day during the first two weeks of life, while SHAM treated pups instead received PBS. After four weeks of rest, the now adult mice were given a single dose of OVA in order to induce OVA-specific oral tolerance. Tolerance was measured in a food allergy model [18]. In this model, mice are sensitized with OVA-alum and challenged by repeated OVA feeding. This induces gut hypersensitivity with infiltration of mast cells and eosinophils into the intestinal mucosa, diarrhea, and elevated concentrations of eosinophils in the blood.

As seen in figure 1, mice that had been neonatally treated with SEA, and thereafter fed a tolerizing dose of OVA, were profoundly protected against OVA-mediated hypersensitivity in the food allergy model. These mice had reduced infiltration of mast cells and eosinophils into the gut, diarrhea and blood eosinophilia, not only compared with PBS fed controls, but also compared with OVA-tolerized mice that were SHAM treated as newborns (i.e. had not been neonatally immune stimulated by SEA) (Fig. 1B–E). OVA tolerization reduced OVA-specific IgE and total IgE by approximately 90%, but no further reduction was seen in neonatally SEA treated mice (data not shown). Thus, neonatal SEA treatment augmented the capacity to develop oral tolerance to a novel dietary protein fed in adult age. It should be noted that improved protection against food allergy was only seen in mice given a tolerizing dose of OVA. Mice that had not been orally tolerized were equally hypersensitive to OVA, regardless of whether they had received SEA or not during infancy (Fig. 1B–E). Thus, neonatal SEA treatment was protective by specifically enhancing the capacity to develop oral tolerance and this capacity lasted long after the mice had received their immunostimulating treatment.

At the time of OVA feeding, we noted no alterations in major lymphocyte subsets, or in their activation or memory markers. These observations have been published [16]. Staphylococcal enterotoxins bind to and activate T cells with certain T cell receptor Vβ regions. Anergy or deletion of these T cells has been observed after parenteral superantigen exposure [19], [20]. Such anergy may persist several weeks after treatment [21]. To exclude that this was the cause of the improved oral tolerance observed in SEA treated mice, we studied *in vitro* proliferation of splenocytes directly (3 h) after the last SEA treatment at two weeks of age, and at six weeks of age, i.e. when the mice showed improved capacity to develop tolerance to OVA. Directly after SEA treatment, responsiveness to SEA was blunted while the responsiveness to the mitogen ConA was increased (P = 0.03 for both comparisons, figure S1). However, at six weeks of age, there was no evidence of T-cell anergy, since splenocytes proliferated equally well to ConA or SEA, regardless of whether the mice had been neonatally immune stimulated or not (figure S1). We also examined signs of Vβ-repertoire skewing. In mice, SEA reacts with T cells bearing TCR Vβ1, Vβ3, Vβ10, Vβ11, Vβ12 and Vβ17 [22], and we have previously reported that mice neonatally exposed to SEA have a tendency towards a lower proportion of Vβ11 specific CD4^+^ T cells at six weeks of age [16]. Here, we examined the TCRVβ repertoire of CD4^+^ T cells in general (figure S2), as well as CD4^+^FoxP3^+^ T cells and CD8^+^ T cells (data not shown), both in two weeks and six weeks old mice. At two weeks of age, the most striking effect of the Vβ repertoire was an increased proportion of Vβ3^+^ T cells and a decreased proportion of Vβ2^+^ T cells in SEA relative to SHAM treated mice (figure S2). At six weeks, SEA treated mice had, as we showed previously, a slightly reduced proportion of Vβ11^+^ T cells and a slightly increased proportion of Vβ8.3, as compared to SHAM treated mice. In general, at six weeks of age, which is the time when we induce oral tolerance, we observed very limited effects on the Vβ-repertoire (figure S2). The levels of dead and apoptotic cells were also determined at six weeks of age in neonatally SEA or SHAM treated mice (gating strategy is shown in figure S3A–E). The proportions of necrotic, apoptotic, and live cells in the CD4^+^ and CD8^+^ T-cell populations, did not differ between SEA and SHAM treated mice (figure S3F–G). Neither did the proportion of CD4^+^ and CD8^+^ T cells differ between neonatally superantigen treated and control mice (figure S3H).

Thus, at the time point when the neonatally SEA treated mice were more prone to develop oral tolerance, neither anergy, nor deletion of the whole CD4^+^ or CD8^+^ T cell subsets, could explain the improved oral tolerance observed in the SEA treated mice.

### Neonatal SEA treatment leads to increased expression of FoxP3 among transferred OVA-specific T cells

**Figure 2:**
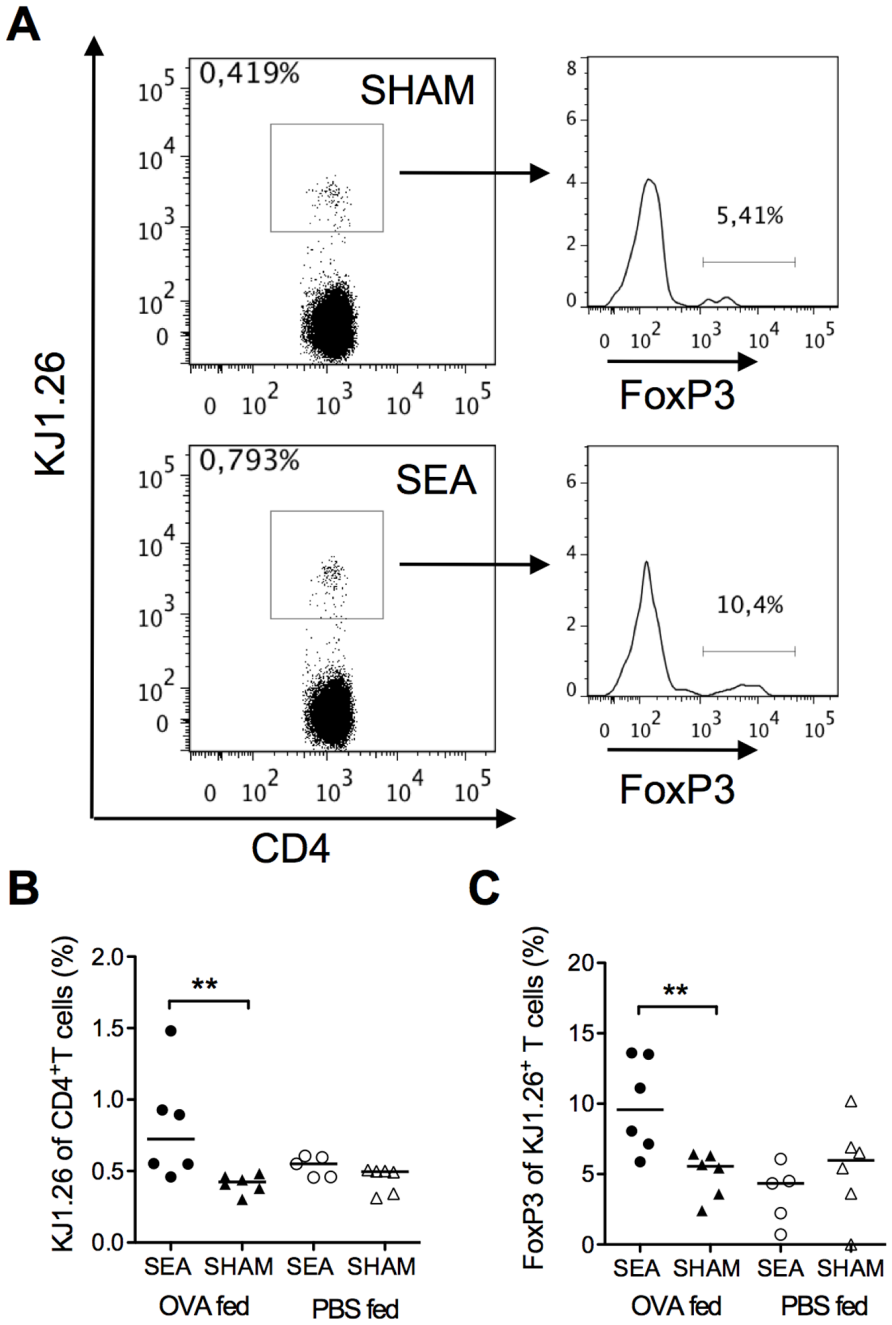
Neonatally SEA-treated mice exhibit a stronger OVA-specific response after oral tolerization with OVA. Mice (n = 5–6) were fed staphylococcal enterotoxin A (SEA) or PBS (SHAM) perorally on six occasions during the first two weeks of life. At six weeks of age all mice were injected i.v. with 1×10^6^ OVA-specific DO11.10 CD4^+^ T cells. At 8 h post cell transfer mice were fed 5 mg OVA or PBS and at 15 h later all mice were sacrificed and mesenteric lymph nodes (MLN) were collected for flow cytometric analysis. MLN cells were stained for surface expression of CD4, and the OVA-specific T cell marker KJ1.26 and for intracellular FoxP3. (A) Representative dot plots of MLN cells stained with CD4 and KJ1.26 and histograms of the CD4^+^KJ1.26^+^ population showing FoxP3. (B) Proportion of KJ1.26^+^ of CD4^+^ T cells. (C) Proportion of FoxP3^+^ of KJ1.26^+^CD4^+^ T cells. In B–C, each symbol represents one animal and the horizontal line shows median value of the group. Data are representative from two independent experiments. ** P<0.01, analyzed with Mann-Whitney U-test.

Oral tolerance is thought to be mediated by induced Tregs [7]. We hypothesized that the conditions prevailing in the gut of adult mice that had received SEA neonatally favored maturation of Tregs to novel antigens introduced perorally. We injected OVA-specific CD4^+^ T cells, obtained from transgenic DO11.10 mice, into six weeks old mice (given SEA, or SHAM treatment, as neonates). Recipients were fed a tolerizing dose of OVA and the fate of the transferred OVA-specific CD4^+^ T cells was examined by flow cytometry at 15 h after transfer. A representative gating for the DO11.10 cell marker KJ1.26 is shown in figure 2A. In mice that had been SEA exposed neonatally, feeding of OVA induced expansion of OVA-specific T cells (P = 0.009, Fig. 2B) and a larger proportion of them expressed FoxP3 (P = 0.009, Fig. 2C), than in OVA-fed mice not neonatally exposed to SEA.

### Increased ALDH activity in MLN DCs of neonatally SEA exposed mice

**Figure 3:**
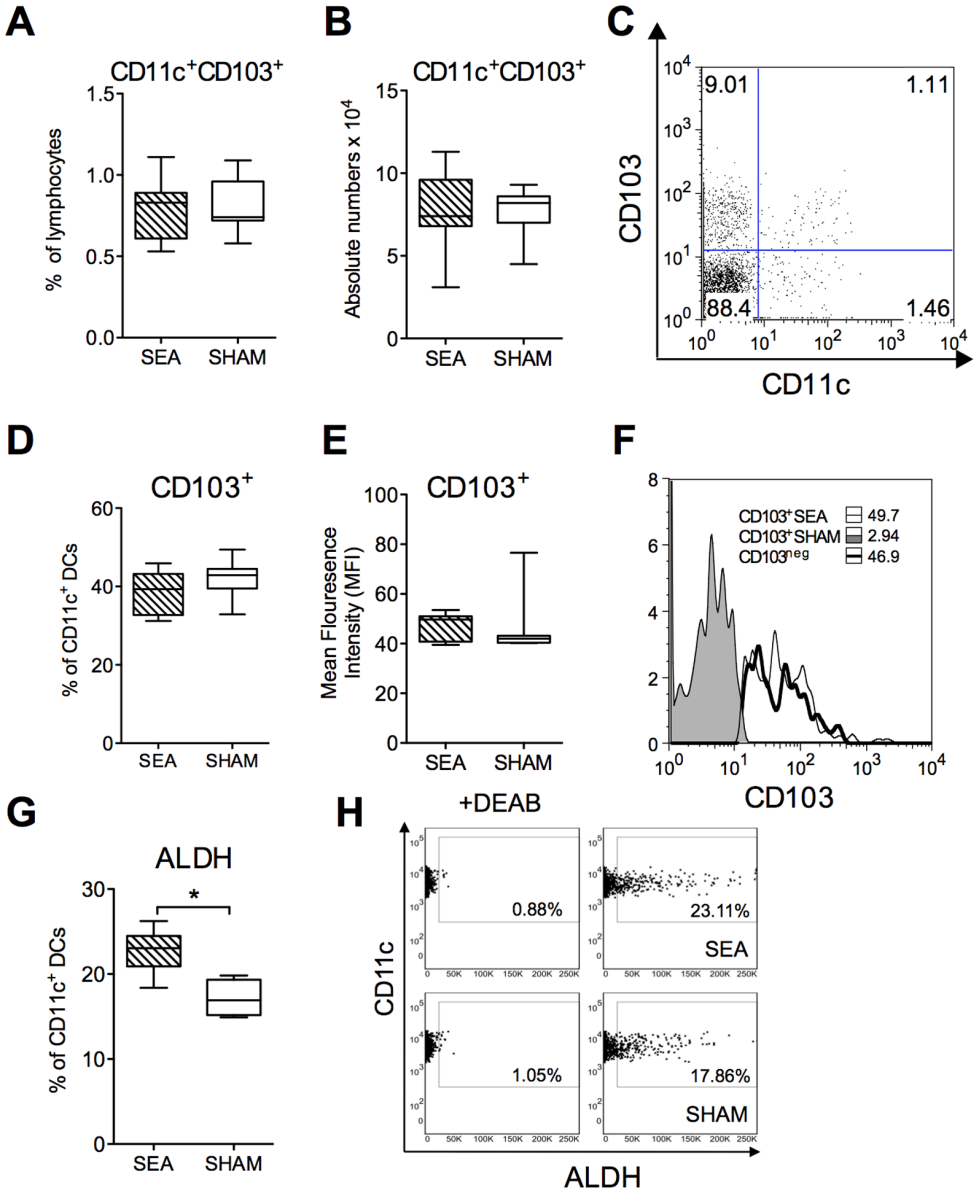
Increased expression of ALDH by CD11c^+^ DCs after neonatal SEA treatment. Mice (n = 6 in each group) were fed staphylococcal enterotoxin A (SEA) or PBS (SHAM) perorally on six occasions during the first two weeks of life. Mesenteric lymph nodes (MLNs) were collected at four weeks after treatment and the cells were subjected to flowcytometric analysis. A) The proportion of CD11c^+^CD103^+^ DCs among live-gated MLN cells. B) The absolute number of CD11c^+^CD103^+^in MLN. C) gating of CD11c^+^ CD103^+^ DCs in MLN. D) Proportion of CD103^+^ cells among the CD11c^+^ DC population. E) Mean Fluorescence Intensity (MFI) of CD103 on the CD11c^+^ DC population. F) Representative histogram of CD103 expression on CD11c^+^DC population. The ALDEFLUOR® assay was performed in order to identify CD11c^+^ cells with aldehyde dehydrogenase (ALDH) expression (ALDH^+^). The ALDH inhibitor diethylaminobenzaldehyde (DEAB) was used as a negative control. The cells without inhibitor, shifted to the right, were considered ALDH+ cells. (G) The proportion of CD11c+ cells expressing ALDH. (H) Representative dot plot of MLN cells stained with CD11c and ALDH (right panel) and with DEAB (left panel). Error bars represent SD and horizontal line shows the median value of the group. Data are representative from two independent experiments. * P<0.05, analyzed with Mann-Whitney U-test.

It appeared that FoxP3 was expressed on CD4^+^ T cells with a greater efficiency in adult mice that had been exposed to SEA neonatally. Conversion of naïve T cells into Tregs can be carried out by CD103^+^ DCs, which are prevalent in the gut mucosa and in the MLN. Conversion depends on the capacity of this DC subset to produce RA from vitamin A, a reaction catalyzed by the RALDH enzyme. We analyzed whether neonatally SEA treated mice had increased proportions, or numbers, of CD103^+^ DCs when reaching adult age and/or whether their RALDH expression was increased. In six weeks old mice, the proportion and numbers of CD11c^+^CD103^+^ DCs in the MLN did not differ depending on neonatal SEA exposure (Fig. 3A–B, gating is shown in fig. 3C). The expression of CD103, measured as mean fluorescence intensity (MFI), or as the proportion of CD103+ cells among the CD11c^+^ DCs, were also comparable between neonatally SEA and SHAM treated mice (Fig. 3D–E, MFI histogram is shown in fig. 3F). However, mice that had been exposed to SEA neonatally displayed an increased proportion of CD11c^+^ DCs expressing the RALDH enzyme (P = 0.02, Fig. 3G, gating is shown in fig. 3H). Thus, an enhanced capacity to generate retinoic acid by the CD11c^+^ DC population might explain the increased expression of FoxP3 among stimulated Ag-specific CD4^+^ T cells, after oral tolerization, in mice that had received a neonatal immune stimulation by SEA.

### DCs from neonatally SEA exposed mice have increased capacity to imprint FoxP3 and α4β7 on T cells *ex vivo*

**Figure 4:**
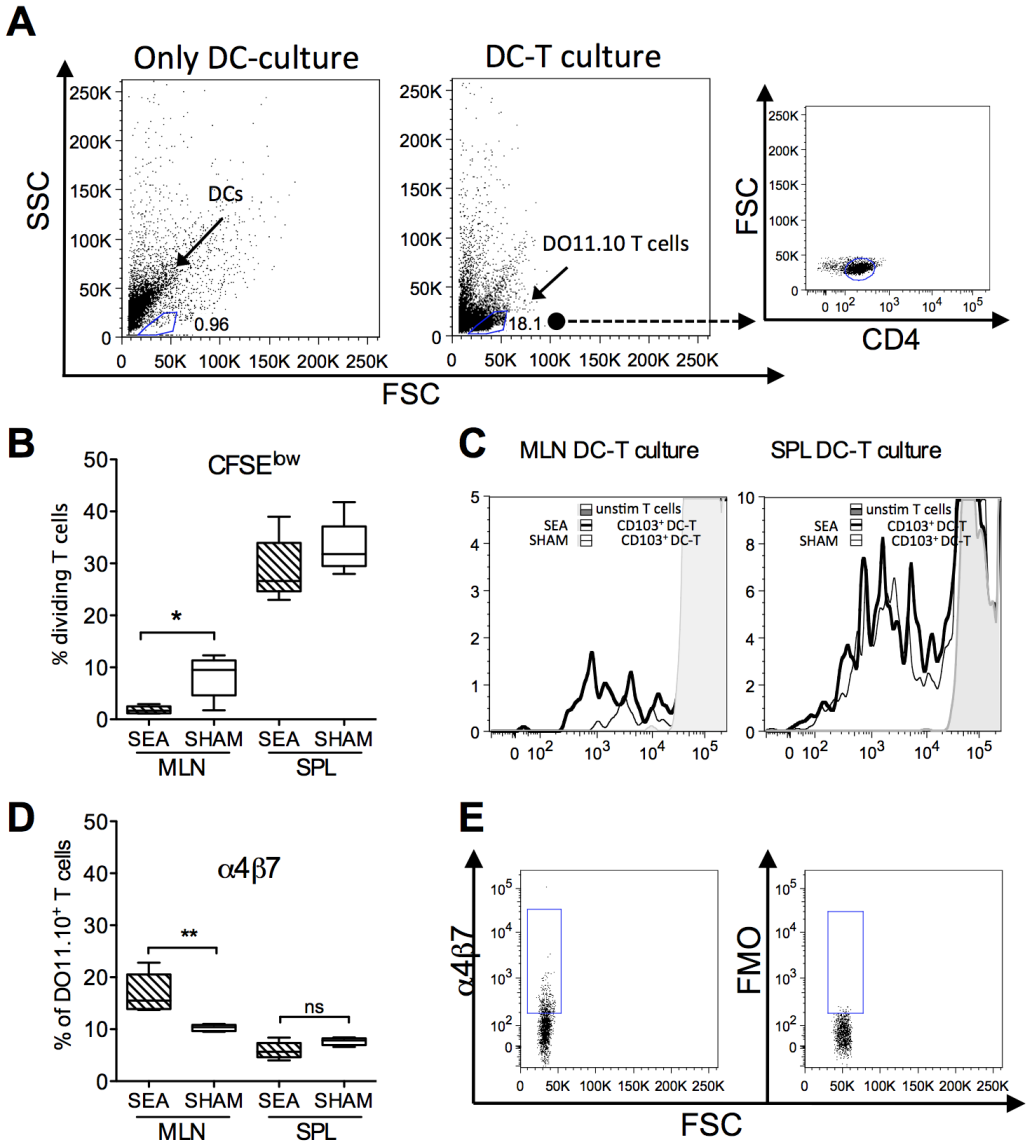
CD11c^+^ DCs from neonatally SEA treated mice exhibit enhanced gut-imprinting ability *in vitro*. Mice (n = 6 in each group) were fed staphylococcal enterotoxin A (SEA) or PBS (SHAM) perorally on six occasions during the first two weeks of life. Mesenteric lymph nodes (MLNs) and spleens were collected at six weeks of age and CD11c^+^ cells were enriched by positive MACS isolation. The DCs were pulsed with OVA and co-cultured with OVA-specific CFSE stained DO11.10 CD4^+^ T cells. Proliferation, as estimated by reduced CFSE intensity and α4β7 expression were determined in KJ1.26^+^CD4^+^ T cells, by flow cytometry, after six days of culture. (A) Representative gating of cultured T cells. Left plot shows DCs cultured alone, middle plot shows gating of T cells cultured with DCs and right plot show expression of CD4 on gated cells. (B) Proportion of dividing cells with reduced CFSE intensity in MLN and spleen. (C) Representative histogram of CFSE intensity on gated CD4 cells co-cultured with DCs from MLN (left plot) and spleen (right plot). Light gray histogram represents unstimulated CD4 cells, thick line, cultures with DCs from SEA mice and thin line, cultures with DCs from SHAM mice. D) Proportion of α4β7 expressing CD4^+^ T cells in co-cultures with T cells and DCs from MLN and spleen. E) Representative gating of α4β7 (left) and control (FMO, right) of cultivated CD4^+^ T cells. Error bars represent SD and the horizontal line shows the median value of the group. Data shown are representative of two independent experiments. * P<0.05 and** P<0.001, analyzed with Mann-Whitney U-test.

**Figure 5:**
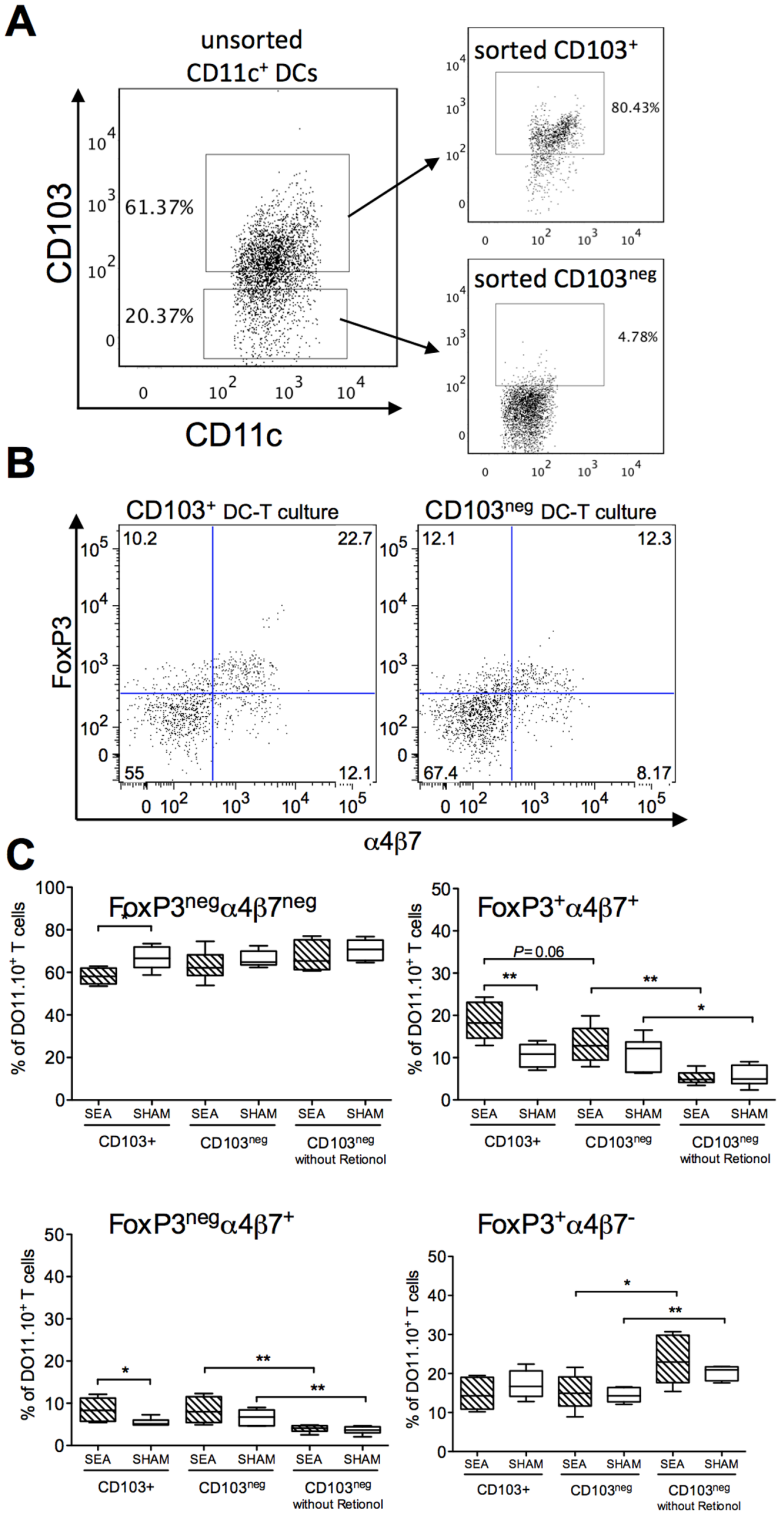
CD103^+^ DCs from neonatally SEA treated mice exhibit enhanced gut-imprinting and FoxP3-inducing ability in vitro. Mice (n = 6 in each group) were fed staphylococcal enterotoxin A (SEA) or PBS (SHAM) perorally on six occasions during the first two weeks of life. Mesenteric lymph nodes (MLNs) were collected at four weeks after treatment and CD103^+^CD11c^+^and CD103^neg^CD11c^+^cells were separated by CD11c-positive MACS isolation followed by sorting for CD103^+^ cells on a flow cytometer. The DCs were pulsed with OVA and co-cultured with OVA-specific DO11.10 CD4^+^ T cells in medium containing 100 nM retinol. FoxP3 and α4β7 expression was determined in KJ1.26^+^CD4^+^ T cells, by flow cytometry, after six days of culture. (A) Representative dot plots of unsorted and CD103-sorted CD11c^+^ DCs. (B) Representative dot plot and gating strategy of the CD4^+^KJ1.26^+^ T cells. C) Proportion of FoxP3^neg^ α4β7^neg^, FoxP3^+^ α4β7^+^, FoxP3^neg^ α4β7^+^and FoxP3^+^ α4β7^neg^ cells among the CD4^+^KJ1.26^+^ T cells cultivated with CD103^+^DCs, CD103^neg^ DCs and CD103^neg^DCs in cultures without Retinol. Error bars represent SD and horizontal line shows the median value of the group. Data shown are representative of two independent experiments. * P<0.05, analyzed with Mann-Whitney U-test.

To investigate the functional capacity of the CD11c^+^ DC subsets in adult mice treated with SEA neonatally, we started off by collecting the whole CD11c^+^ population from the MLN and spleen, by using magnetic beads coated with α-CD11c. CD11c^+^ DCs were pulsed with OVA and co-cultured with CFSE stained OVA-specific DO11.10 T cells. We assessed expression of the gut homing marker α4β7 and proliferation, the latter estimated as decrease of CFSE intensity. As DCs cultivated *in vitro* display quite prominent autofluorescence, proper gating of T cells required wells containing only DCs, which enabled us to separate DCs from T cells; representative gating strategy is shown in figure 4A. We found that CD11c^+^DCs from the MLN were quite poor inducers of T cell proliferation, as compared to CD11c^+^ DCs from the spleen. DCs from MLN stimulated about 10% of T cells, whereas DCs from the spleen stimulated about 30% of T cells (Fig. 4B–C). Moreover, DCs from the MLN of neonatally SEA treated mice induced less T cell proliferation as compared to MLN DCs from SHAM mice (P = 0.03, Fig. B–C). However, DCs from SEA mice were able to imprint α4β7 on a larger proportion of the cultivated T cells, compared to DCs from SHAM mice (P = 0.008 Fig. 4D, representative gating is shown in 4E). Spleen DCs induced α4β7 expression on about 10% of cultivated T cells with no difference between DCs from SEA or SHAM treated mice (Fig. 4D). As gut-imprinting capacity has been ascribed to CD11c^+^CD103^+^ DCs, we isolated this DC subset, by FACS sorting (Fig. 5A), pulsed the cells with OVA, and co-cultured them with OVA-specific DO11.10 T cells. We also added retinol, the substrate for RALDH, to the DC-T cell cultures. Expression of the gut homing marker α4β7 and the Treg marker FoxP3 was examined in the T cells after co-culture. We found that CD103^+^ DCs obtained from neonatally SEA treated mice were more prone to induce FoxP3 and α4β7 expression upon the T cells compared to corresponding DCs from SHAM-treated mice i.e. a larger proportion of stimulated T cells co-expressed FoxP3 and α4β7 (P = 0.005, Fig. 5C). Consequently, a smaller proportion of cells expressing neither FoxP3 nor α4β7 were observed in these cultures (P = 0.01, Fig. 5C). A higher proportion of cells expressing α4β7 alone were noted in cultures with CD103^+^ DCs from SEA mice, compared to corresponding DCs from SHAM mice (P = 0.02), while there was no difference in the proportion of cells expressing FoxP3 alone (Fig. 5C). Surprisingly, the CD103^neg^ DC population also induced FoxP3 and α4β7 expression upon stimulated T cells to a similar extent as did CD103^+^ DCs isolated from SHAM mice (Fig. 5C). The ability of CD103^neg^ DCs to imprint gut homing markers was lost when cultivation was performed without retinol, while induction of FoxP3 was retained (Fig. 5C). To summarize, CD103^+^ DCs, isolated from neonatally SEA treated mice, had improved gut homing/Treg imprinting ability, as compared to CD103^+^ DCs isolated from SHAM treated mice. CD103^neg^ DCs could induce gut homing markers in the presence of retinol, but their ability to do so did not differ between SEA and SHAM treated mice. Hence, it seemed as neonatal SEA exposure increased the functional capacity of CD103^+^DCs, but not the CD103^neg^ DCs.

An increased capacity of the CD103^+^ DC subset to imprint gut homing markers on T cells could explain our previous finding of increased expression of gut homing markers on CD4^+^FoxP3^+^ Tregs in adult mice exposed to SEA neonatally [16]. Here, we confirmed these findings by examining MLN T-cell populations in six weeks old mice. CD4^+^ T cells more often expressed the gut homing marker α4β7 in mice that had been neonatally SEA treated compared with SHAM treated mice (P = 0.03) and the same was true for CD4^+^FoxP3^+^ cells (P = 0.04, Fig. S4). Increased imprinting of gut homing marker CCR9 on B cells from MLN was also evident in mice that had been neonatally SEA treated compared with SHAM treated mice (P = 0.03, Fig. S4).

### Neonatal SEA treatment leads to increased FoxP3^+^Treg density in the gut mucosa

**Figure 6:**
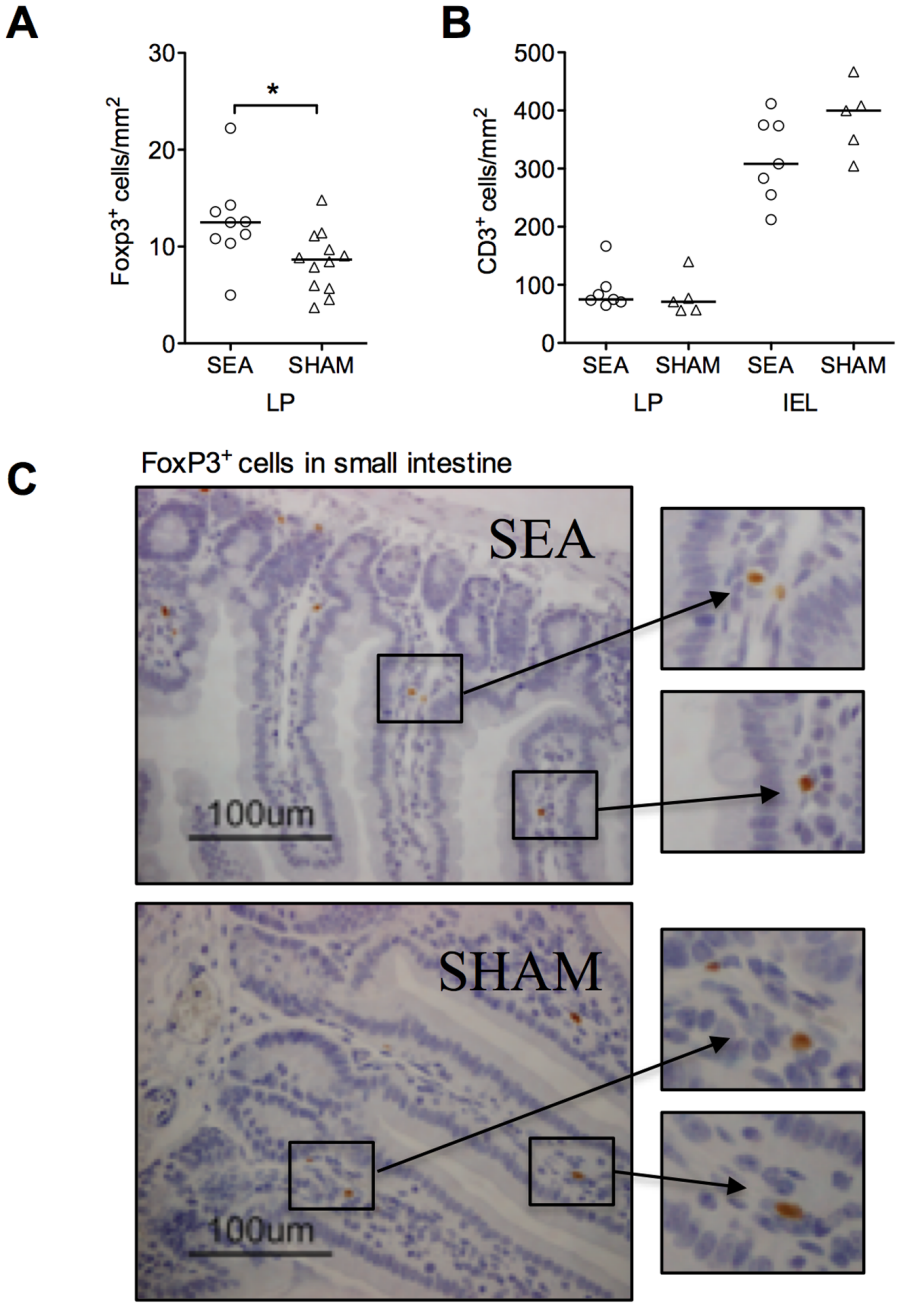
Neonatal SEA-treatment leads to an accumulation of FoxP3+ cells in the gut of mice at adult age. Mice (n = 6 in each group) were fed staphylococcal enterotoxin A (SEA) or PBS (SHAM) perorally on six occasions during the first two weeks of life. At four weeks after treatment, pieces of the small intestine were collected, formalin-fixed, paraffin-embedded and stained for FoxP3 or CD3 by immunohistochemistry. (A) Number of FoxP3^+^ cells/mm^2^. (B) Number of CD3^+^ T cells/mm^2^ in the LP and between epithelial cells, representing intraepithelial lymphocytes (IEL). (C) Representative histological sections of small intestine stained with FoxP3, scale bar = 100 µm. Data shown are representative of two independent experiments. Each symbol represents one animal and horizontal line shows median value of the group. *P<0.05, analyzed with Mann-Whitney U-test.

Increased expression of gut homing markers may enable CD4^+^ T cells, including FoxP3^+^ Tregs to circulate via the gut mucosa. Interestingly, such gut homing has been shown to be a prerequisite for oral tolerance [7]. We examined gut sections of six weeks old mice for FoxP3^+^ cells as well as T cells in general (CD3^+^) (Fig. 6). Indeed, mice that had been treated with SEA neonatally had increased density of FoxP3^+^ cells in the small intestinal lamina propria compared to neonatally SHAM-treated mice (P = 0.02, Fig. 6A, representative histological picture is shown in figure 6C). The density of CD3^+^ T cells in general was neither increased in the lamina propria or within the epithelium (Fig. 6B). We also examined gut sections for the density of eosinophils and mast cells but none of these cell populations were altered in six weeks old mice that had been immune stimulated neonatally (data not shown).

### Inhibition of ALDH blunts oral tolerance development, reduces mucosal Treg and MLN CD103^+^ DCs

The CD103^+^ DC subset might be central in oral tolerance as they mediate conversion of naïve T cells into Tregs. Our results suggest that neonatal superantigen treatment improve the functional capacity of this cell subset via up-regulation of their Vitamin A converting enzyme RALDH. We examined the role of functional Vitamin A conversion on the improved oral tolerance seen in adult mice that had received neonatal immune stimulation by blocking RALDH activity in vivo. The ALDH inhibitor Citral was added to the drinking water between four and six weeks of age of mice that had, or had not, received neonatal SEA stimulation. At six weeks of age they were fed a tolerizing dose of OVA and oral tolerance was measured as protection in the OVA food allergy model.

**Figure 7:**
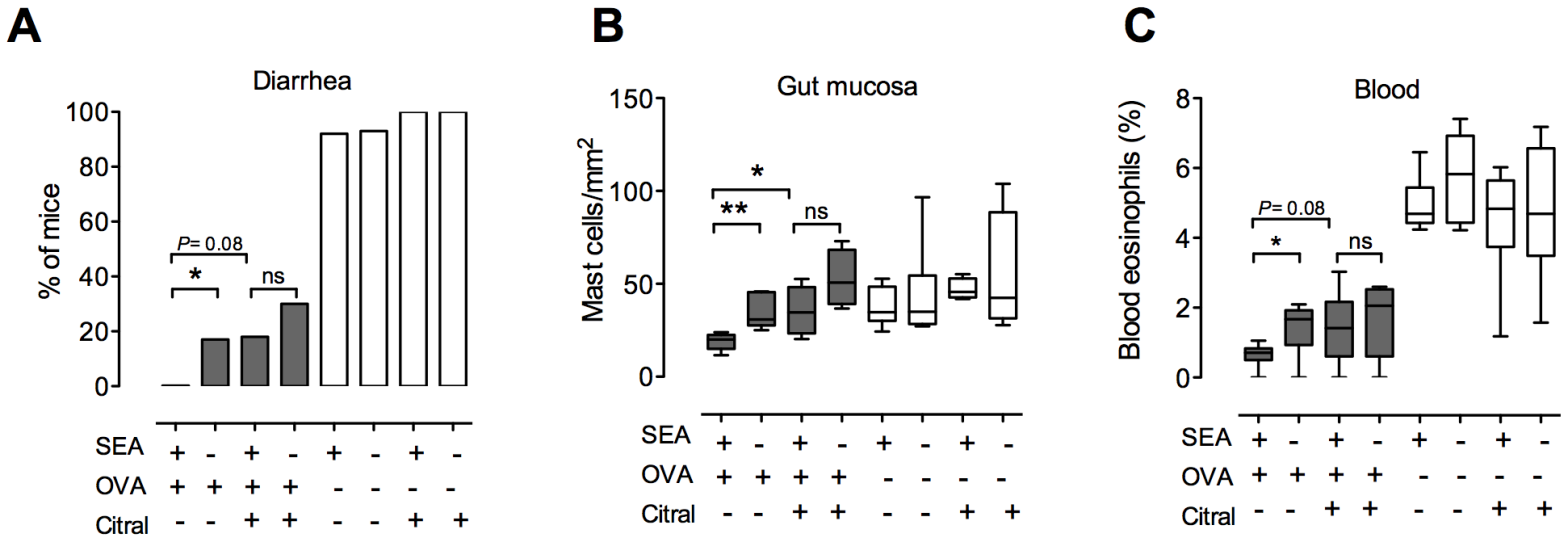
Reduction of ALDH activity by citral affects oral tolerance development. Mice were fed staphylococcal enterotoxin A (SEA) or PBS (SHAM) perorally on six occasions during the first two weeks of life. Two weeks after treatment, groups of mice were given the aldehyde dehydrogenase (ALDH) inhibitor citral in the drinking water. Oral tolerance was induced ten days after citral intake by feeding OVA. After oral tolerization, food allergy was induced by sensitization through two i.p. injections of alum-adsorbed OVA followed by intragastrical (i.g.) challenge with OVA. (A) Mice (n = 5–7 in each group) were monitored for onset of diarrhea, and the percentage of diarrhea positive mice after each challenge was estimated. Blood and pieces of the small intestine were collected for analysis after the final challenge. (B) Number of mast cells in the small intestine was estimated in tissue sections. (C) Proportion of eosinophils in the blood was estimated by differential counts of blood cells. Data shown are representative of two independent experiments. * P<0.05 and ** P<0.01, analyzed with Fisher's exact test (onset of diarrhea) or Mann-Whitney U-test.

The results are shown in figure 7. Again, we found improved oral tolerance to OVA in mice that had been given SEA neonatally, but this effect was abolished if the mice were fed Citral during the time when the tolerizing OVA dose was administered. Improved oral tolerance was measured as reduced infiltration of mast cells into the gut, diarrhea and blood eosinophilia (Figure 7A–C). The results imply that the elevated ALDH activity characterizing adult mice that had been immunostimulated neonatally could be involved in the improved oral tolerance observed in these animals.

**Figure 8:**
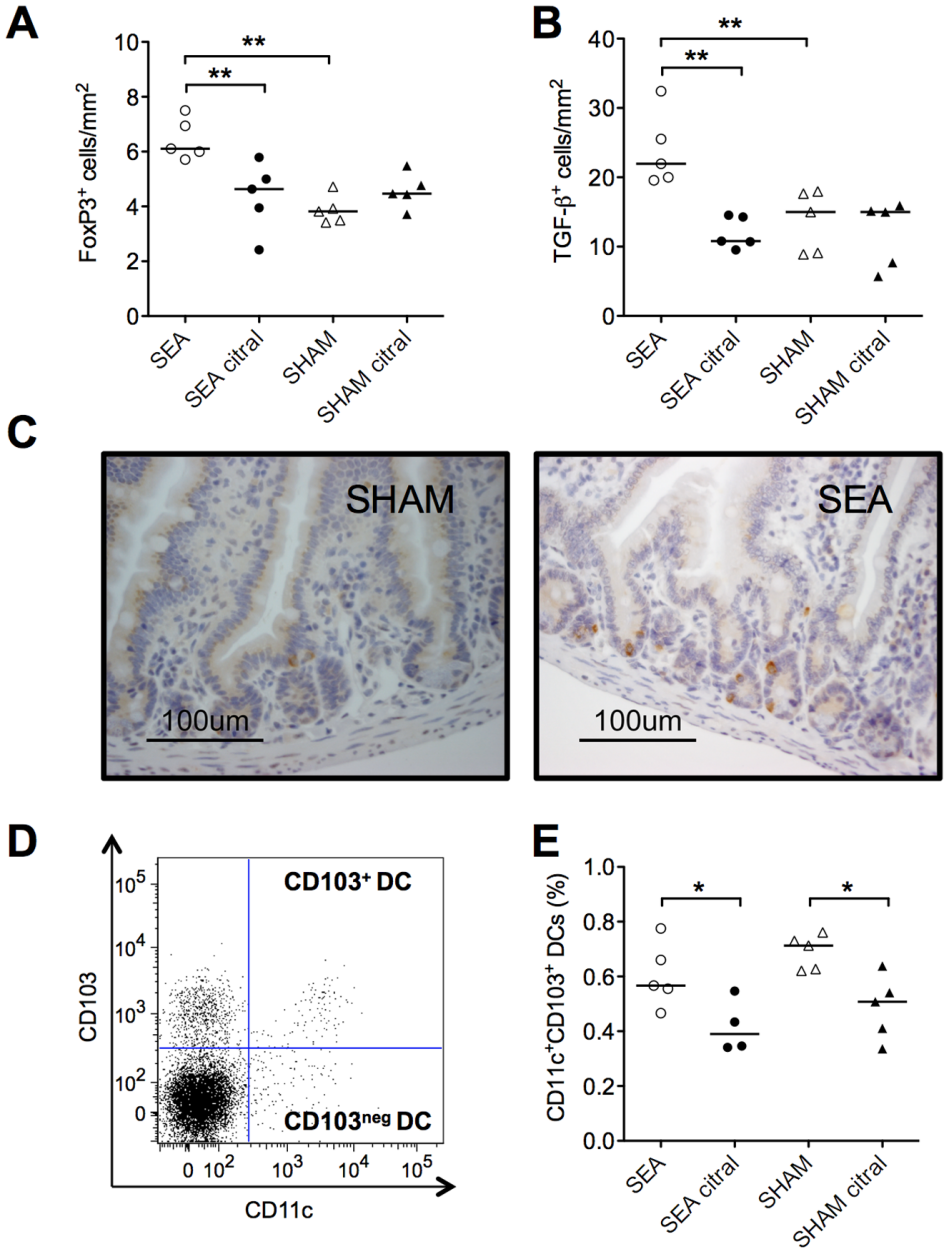
Reduction of ALDH activity by citral abolishes FoxP3 and TGF-β accumulation in the gut and diminishes CD103^+^ DCs in the MLN. Mice were fed staphylococcal enterotoxin A (SEA) or PBS (SHAM) perorally on six occasions during the first two weeks of life. Two weeks after treatment, groups of mice were given the aldehyde dehydrogenase (ALDH) inhibitor citral in the drinking water. Formalin-fixed, paraffin-embedded sections of the small intestine were collected after ten days of citral intake and analyzed by immunohistochemistry for FoxP3 and TGF-β (n = 5 in each group). Mesenteric lymph nodes (MLN) were collected and stained for flow cytometric analysis of CD11c and CD103. (A) Number of FoxP3^+^ cells/mm2, (B) number of TGF-β^+^ cells/mm^2^ and (C) representative histological sections of small intestine stained with TGF-β, scale bar = 100 µm. (D) Representative dot plot of CD11c^+^CD103^+^ gating strategy of live cells and (E) proportion of CD11c^+^CD103^+^ DCs. Each symbol represents one animal and horizontal line shows median value of the group. Data shown are representative of two independent experiments. * P<0.05 and ** P<0.01, analyzed with Mann-Whitney U-test.

We also examined the effect of Citral treatment on the density of FoxP3^+^ cells in the intestinal lamina propria. As shown in figure 8A, neonatally SEA treated mice again had a higher density of FoxP3^+^ cells in the gut mucosa at six weeks of age compared to SHAM-treated mice (P = 0.008). However, this increase was not seen in animals fed Citral between four and six weeks of age. The same was true for TGF-β-producing cells (P = 0.008; Fig. 8B). For representative histological sections of TGF-β, see figure 8C. In addition, Citral treatment led to a decreased proportion of CD103^+^ DCs in the MLN (P = 0.03; Fig. 8E), whereas the proportion of CD11c^+^CD103^neg^ DCs was unaffected (data not shown). The gating strategy is shown in figure 8D.

## Discussion

According to the hygiene hypothesis, immune stimulation early in life is needed to confer protection against allergy development later in life [1] and a complex gut microbiota at one week of age reduces the risk of developing allergy [23]. However, no theory has been put forward to explain how an early immune stimulation can affect whether encounter of novel non-microbial antigens later in life results in development of tolerance, or sensitization.

We have treated newborn mice with the staphylococcal superantigen SEA, in an attempt to provide a strong immunostimulatory event early in life. These mice have improved capacity for oral tolerance induction as adults, as shown by increased protection in a model of airway allergy [16]. Here we demonstrate that the improved oral tolerance after neonatal SEA treatment also protects the mice in a food allergy model.

Peroral exposure to Staphylococcal enterotoxins including SEA causes an inflammatory response in the gut mucosa [20] and repeated intravenous administrations of superantigen causes activation of T cells with the corresponding Vβ chain, followed by deletion of these cells both in neonatal [24] and in adult mice [21], [25]–[28]. However, the T-cell levels are restored to normal within a few weeks [29]. To exclude that such effect plays a role in our model, we undertook a quite extensive survey of the immune status of the mice both directly after the last SEA dose and at six weeks or age, which is the time for oral tolerance induction. Directly after SEA treatment, splenocytes were unresponsive towards re-stimulation with SEA *in vitro*, but this effect was no longer present at six weeks of age. Furthermore, there was no general unresponsiveness, as proliferation induced by mitogen was functional also immediately after SEA treatment. The effects on the Vβ repertoire were not dramatic. Directly after the last SEA dose, mice had proportionally more CD4^+^ T cells expressing Vβ3, but at six weeks of age, only subtle differences in Vβ repertoire were seen. We also investigated the levels of live, apoptotic and necrotic cells in the MLN at 4 weeks after treatment with SEA. There were no alterations of those cells in SEA relative to SHAM treated mice, when looking at the whole CD4 and CD8 populations. However, we cannot exclude the possibility that there could be a difference in the levels of live, apoptotic and necrotic cells among the FoxP3^+^ T cells. In addition, there were no signs of unspecific inflammation in the gut mucosa in six weeks old mice that had received neonatal SEA treatment. Thus, anergy, TCR Vβ repertoire skewing, or unspecific inflammation in the gut, were not likely causes of the observed improvement in oral tolerance in adult mice that had received SEA as neonates.

One conspicuous finding was that mice that had been treated neonatally with SEA displayed a larger proportion of T and B cells in the MLN that expressed CCR9 and α4β7, i.e. gut homing markers, compared to SHAM-treated mice. When examining the mucosa of the small intestine of six weeks old mice, we also noted that FoxP3^+^ cells were more numerous in neonatally SEA compared to SHAM treated mice, indicating an increased homing of Tregs to the gut. Interestingly, Hadis et al. has demonstrated that oral tolerance involves antigen-specific expansion of FoxP3^+^ Tregs in the gut lamina propria, a finding that was dependent on the presence of gut homing T cells [7]. Imprinting of gut homing markers on T cells has been ascribed specifically to CD103^+^ DCs, localized in the gut associated lymphoid tissue. These DCs are also known to facilitate the conversion of naïve T cells into induced FoxP3^+^ Tregs [11]–[13].However, we found no evidence of increased numbers of CD103^+^ DCs in the MLN. Both imprinting of a gut homing phenotype on naïve T cells and their conversion into FoxP3^+^ Tregs by the CD103^+^ DC depend on conversion of Vitamin A to retinoic acid via the ALDH enzyme [11]–[13]. We found a higher ALDH activity among CD11c^+^ MLN DCs isolated from SEA, as compared to SHAM treated mice. Further, upon *in vitro* cultivation together with OVA specific T cells, DCs from neonatally SEA treated, adult mice were more prone to induce Tregs with gut homing potential. Accordingly, when mice were fed the ALDH blocker citral from four to six weeks of age, the increased accumulation of FoxP3^+^ cells and TGF-β^+^ cells in the intestinal mucosa of mice treated with SEA neonatally was reduced to background levels, as was tolerance to OVA. Reduced CD103^+^ DC population numbers in the MLN and a lower MHC class II expression on their surface also accompanied this observation. Thus, our results suggest that accumulation of FoxP3^+^ cells in the mucosa and improved oral tolerance persisting for weeks after neonatal immune stimulation depend on an increased functional capacity of CD103^+^ DCs.

The question is how ALDH activity is kept elevated as a “memory” of a strong neonatal immune stimulation. The lifespan of DCs is only counted in days, as is the lifespan of the epithelium. Most likely, this “memory” is carried by memory lymphocytes. One possibility is that T cells imprinted by CD103^+^ DCs are, in turn, able to convert immature CD103^+^ DCs into a more functional phenotype, e.g. by up-regulating their functional ALDH enzyme activity. Thus, gut-homing T cells and CD103^+^ DCs might keep their populations up through a mutual interaction. It is known that Tregs and APCs can endow tolerizing phenotypes on one another as tolerogenic APCs generate Tregs [30]–[32], and Tregs that interact with APCs can render them tolerogenic [33]–[35]. This effect has been linked to the interaction of CD80/CD86 on the APC with CTLA-4 on the Treg, followed by CD80/CD86 removal [36]. However, we found no convincing evidence of a reduced CD80/CD86 expression on the APCs of neonatally SEA-treated mice. It is likely that several mechanisms exist by which Tregs and tolerogenic APCs may interact to enable a tolerogenic state and one of these mechanisms might involve gain of Vitamin A converting capacity by gut DCs.

We do not know why neonatal immune stimulation by SEA leads to increased numbers of Treg in the gut. We have observed that peroral SEA administration improves tolerogenic processing of antigens by the gut epithelium (unpublished data). Further, strong immune stimulation per se, might lead to generation of Tregs. To speculate, some of these Tregs might interact with immature DCs in the gut mucosa and promote their development into a more tolerogenic phenotype.

In summary, our results suggests that improved functional conversion of Vitamin A by the CD103^+^ DC subset might be involved in the augmented oral tolerance seen in animals stimulated neonatally by SEA. The mechanisms by which increased oral tolerance and ALDH activity may persist long after immune stimulation has ceased needs to be further investigated, but mutual interaction between Tregs, and tolerogenic DCs might be pivotal.

## Supporting Information

Figure S1 **In vitro stimulation of splenocytes.** Spleen cell suspensions were prepared from neonatally Staphylococcal enterotoxin A (SEA) treated mice at 2 weeks (15 h after the last SEA/SHAM dose) and at 6 weeks of age (4w after the last SEA/SHAM dose). SEA treated mice were given 5 mg SEA perorally on 6 occasions during the first 2w, SHAM treated mice instead recieved PBS. Splenocytes were suspended in Iscove's complete medium, aliquoted at 1×105 cells/well in microtiter plates and stimulated with 5 mg/mL SEA or 5 mg/ml ConA. Proliferation was measured after 5 days of culture in 5% CO2, at 37°C by 3H-thymidine incorporation during the last 8 h of cultivation. Hatched bars represent spleen cultures from neonatally SEA treated mice, open bars represent spleen cultures from SHAM treated mice. Bars represent mean cpm and error bars represent SEM. * P<0.05, analyzed with Mann-Whitney U-test.
(TIF)

Figure S2 **Determination of TCR Vb-repertoire in splenocytes.** Spleen cell suspensions were prepared from neonatally Staphylococcal enterotoxin A (SEA) treated mice at 2 weeks (15 h after the last SEA/SHAM dose) and at 6 weeks of age (4w after the last SEA/SHAM dose). SEA treated mice were given 5 mg SEA perorally on 6 occasions during the first 2w, SHAM treated mice instead recieved PBS. Splenocytes were stained for CD4 and TCR Vb screening panel according to standard procedure. All cells were acquired using FACSCantoII (BD Biosciences) and analyzed with FlowJo software (Treestar inc., Ashland, OR). Hatched bars represent neonatally SEA treated mice, open bars represent SHAM treated mice. Bars represent mean percentage and error bars represent SEM. * P<0.05, *** P<0.001, analyzed with two-way ANOVA followed by Bonferroni post test.
(TIF)

Figure S3 **Expression of gut homing markers in MLN lymphocytes.** Mice (n = 6–7) were fed staphylococcal enterotoxin A (SEA) or PBS (SHAM) perorally on six occasions during the first two weeks of life. Four weeks after treatment (at 6 weeks of age) mice were sacrificed and mesenteric lymph nodes (MLN) were collected for flow cytometric analyses. Cells were stained for surface expression of CD19, CD4, a4b7 and CCR9 and for intracellular FoxP3. Hatched box represent SEA treated mice, open box represent SHAM treated mice. * P<0.05, analyzed with Mann-Whitney U-test.
(TIF)

Figure S4 **Dead and apoptotic lymphocytes in MLN.** Mice (n = 6) were fed staphylococcal enterotoxin A (SEA) or PBS (SHAM) perorally on six occasions during the first two weeks of life. Four weeks after treatment (at 6 weeks of age) mice were sacrificed and mesenteric lymph nodes (MLN) were collected for flow cytometric analyses. Cells were stained for Annexin V and 7-AAD, according to manufacturers description (BD). Figure A–C demonstrate the gating strategy. A) A quadrant (Annexin V and 7-AAD) was applied on ungated cells. B) The Q4 gate (Annexinneg7-AADneg) was shown in Forward Scatter (FSC) versus Side Scatter (SSC) mode in order to identify debris, C)The debris gate was applied to ungated cells and a non-derbris gate was created. D) CD3+CD8neg (CD4+) and CD8+ was selected from the non-debris gate. E) 7-AAD+AnnexinV+ cells are assumed to be necrotic and dead cells (Necr), 7-AADnegAnnexinV+ early apoptotic cells (Apop) and 7-AADnegAnnexinVneg live cells. Proportion of Annexin V and 7AAD gated cells within the F) CD8+ and G) CD4+ T cells population. H) The proportion of CD4+ and CD8+ T cells in the MLN of SEA and SHAM treated mice.
(TIF)
