# Supplementary figures and images for: Neonatal Mucosal Immune Stimulation by Microbial Superantigen Improves the Tolerogenic Capacity of CD103+ Dendritic Cells
Source: PLoS One. 2013 Sep 27;8(9):e75594. doi: 10.1371/journal.pone.0075594 (PMC3785493; doi:10.1371/journal.pone.0075594)

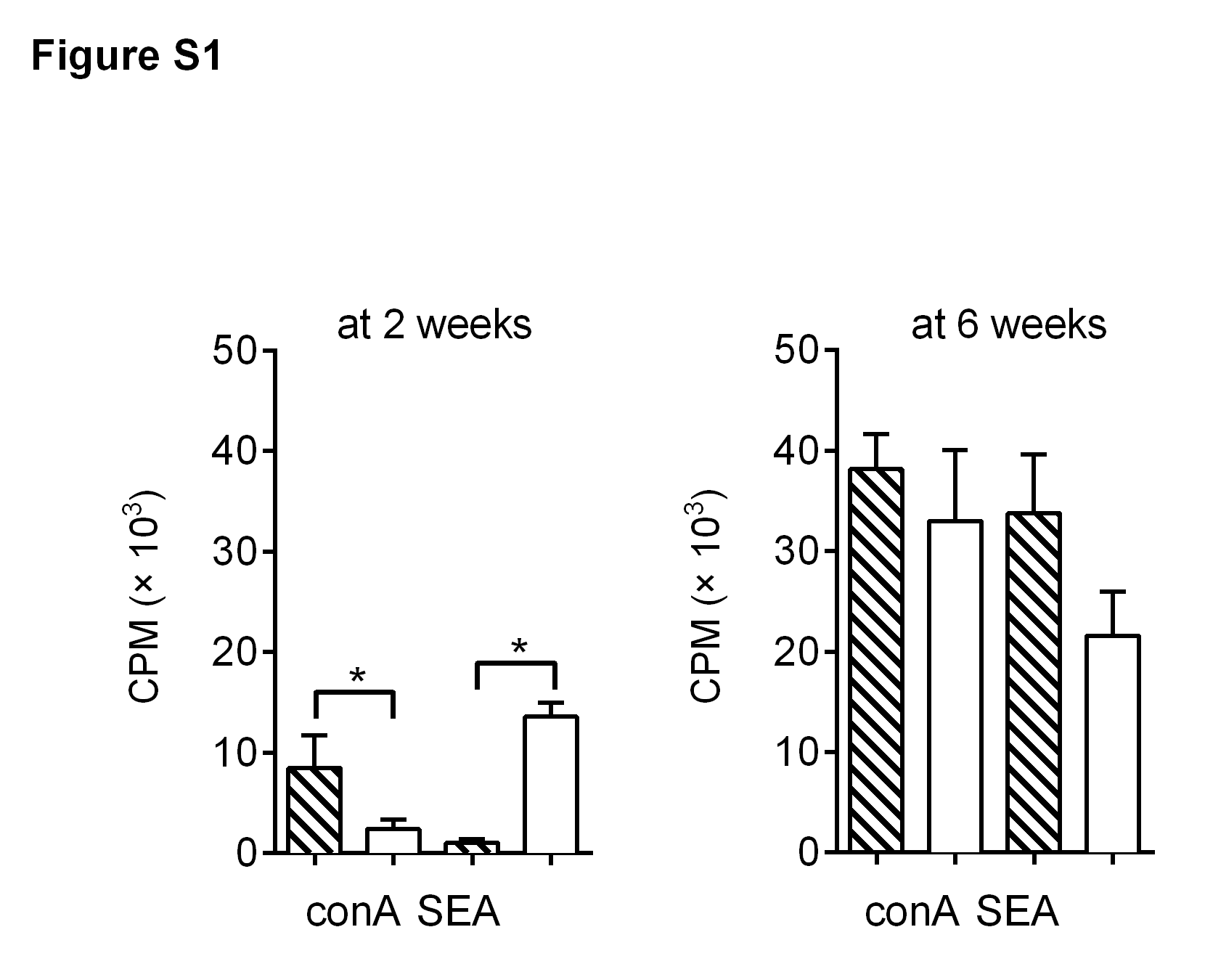

Supplement: Figure S1 — In vitro stimulation of splenocytes. Spleen cell suspensions were prepared from neonatally Staphylococcal enterotoxin A (SEA) treated mice at 2 weeks (15 h after the last SEA/SHAM dose) and at 6 weeks of age (4w after the last SEA/SHAM dose). SEA treated mice were given 5 mg SEA perorally on 6 occasions during the first 2w, SHAM treated mice instead recieved PBS. Splenocytes were suspended in Iscove's complete medium, aliquoted at 1×10^5^ cells/well in microtiter plates and stimulated with 5 mg/mL SEA or 5 mg/ml ConA. Proliferation was measured after 5 days of culture in 5% CO2, at 37°C by 3H-thymidine incorporation during the last 8 h of cultivation. Hatched bars represent spleen cultures from neonatally SEA treated mice, open bars represent spleen cultures from SHAM treated mice. Bars represent mean cpm and error bars represent SEM. * P<0.05, analyzed with Mann-Whitney U-test. (TIF) [file pone.0075594.s001.tif]

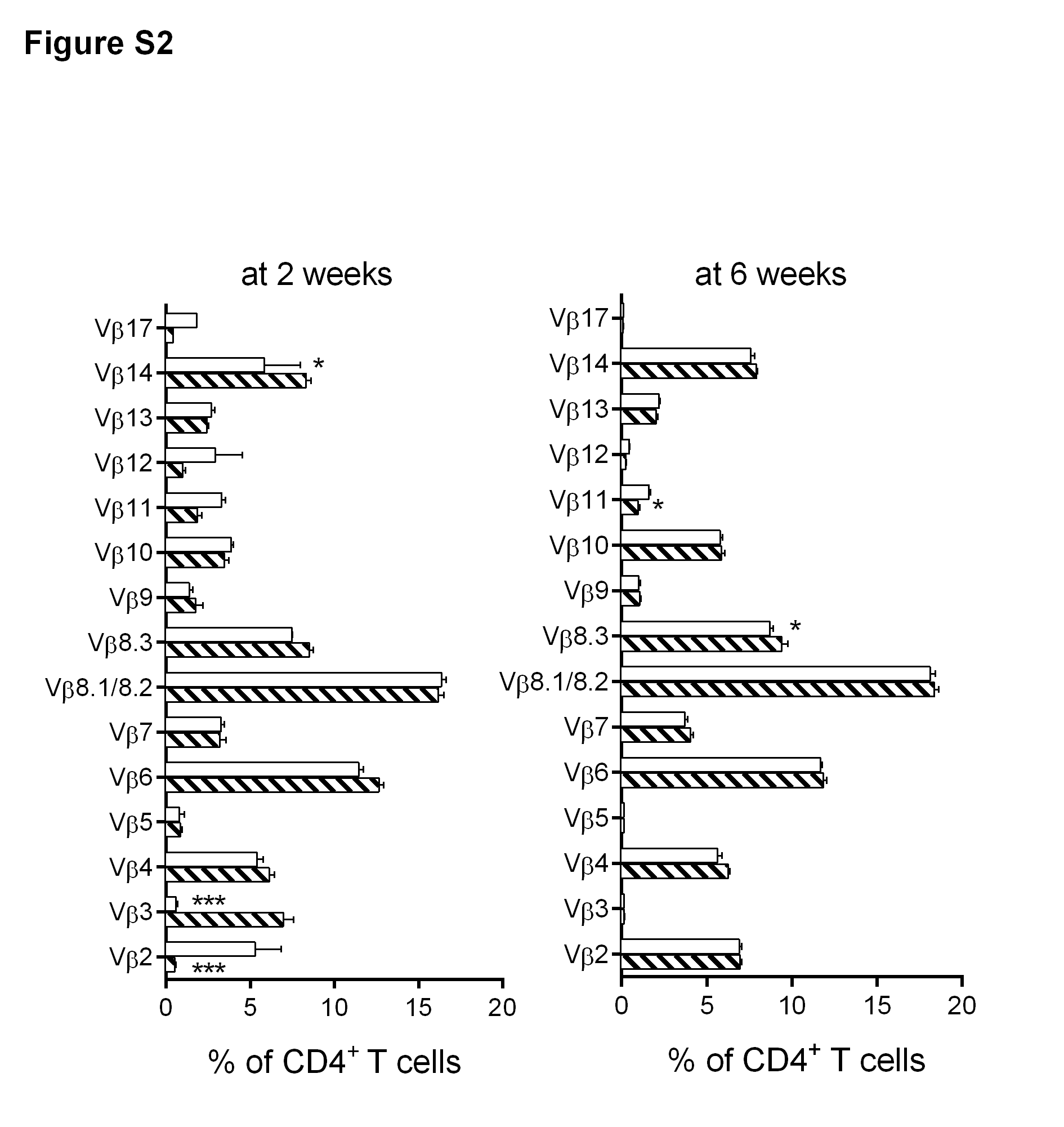

Supplement: Figure S2 — Determination of TCR Vb-repertoire in splenocytes. Spleen cell suspensions were prepared from neonatally Staphylococcal enterotoxin A (SEA) treated mice at 2 weeks (15 h after the last SEA/SHAM dose) and at 6 weeks of age (4w after the last SEA/SHAM dose). SEA treated mice were given 5 mg SEA perorally on 6 occasions during the first 2w, SHAM treated mice instead recieved PBS. Splenocytes were stained for CD4 and TCR Vb screening panel according to standard procedure. All cells were acquired using FACSCantoII (BD Biosciences) and analyzed with FlowJo software (Treestar inc., Ashland, OR). Hatched bars represent neonatally SEA treated mice, open bars represent SHAM treated mice. Bars represent mean percentage and error bars represent SEM. * P<0.05, *** P<0.001, analyzed with two-way ANOVA followed by Bonferroni post test. (TIF) [file pone.0075594.s002.tif]

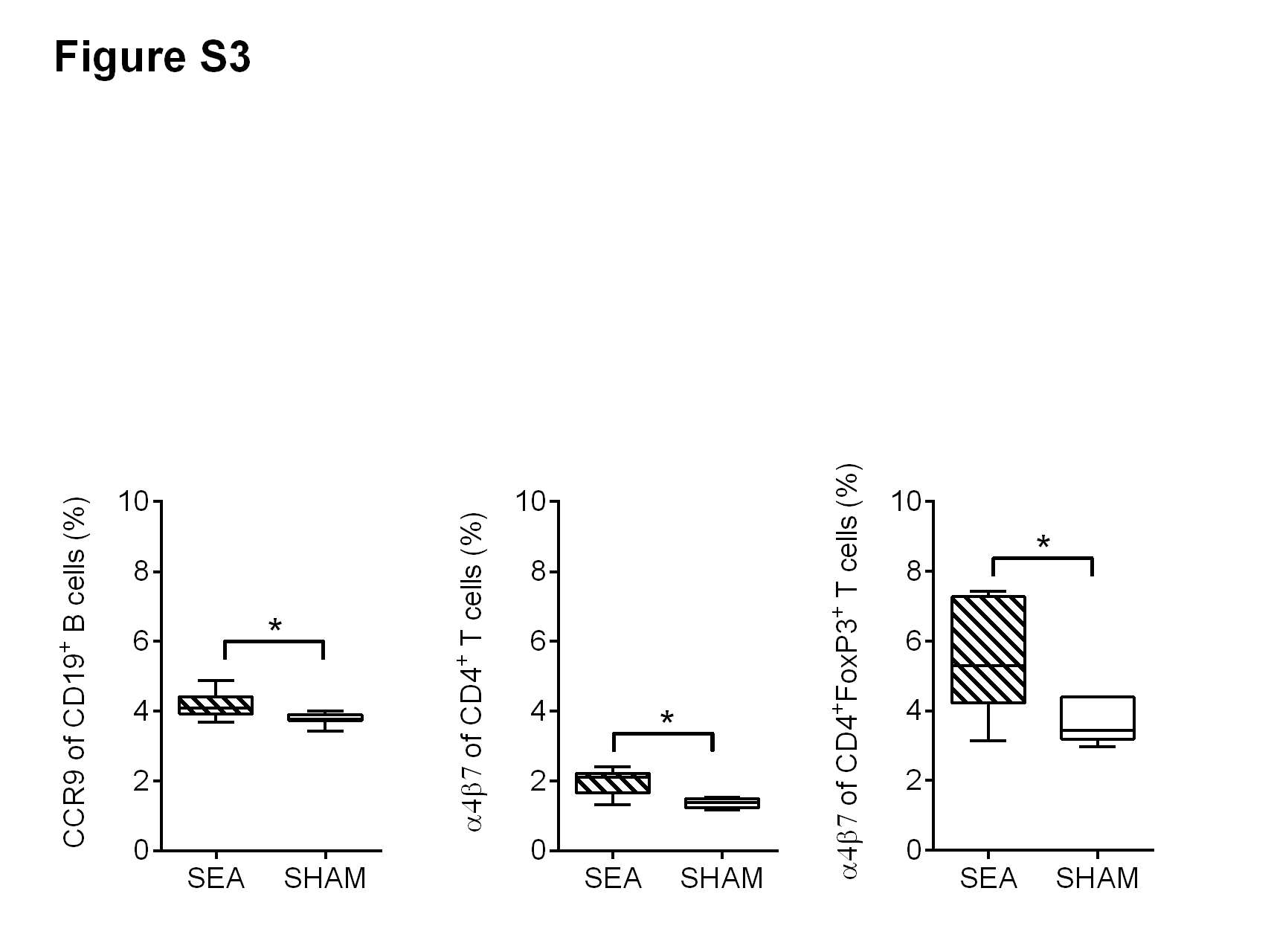

Supplement: Figure S3 — Expression of gut homing markers in MLN lymphocytes. Mice (n = 6–7) were fed staphylococcal enterotoxin A (SEA) or PBS (SHAM) perorally on six occasions during the first two weeks of life. Four weeks after treatment (at 6 weeks of age) mice were sacrificed and mesenteric lymph nodes (MLN) were collected for flow cytometric analyses. Cells were stained for surface expression of CD19, CD4, a4b7 and CCR9 and for intracellular FoxP3. Hatched box represent SEA treated mice, open box represent SHAM treated mice. * P<0.05, analyzed with Mann-Whitney U-test. (TIF) [file pone.0075594.s003.tif]
